# What made us “hunter-gatherers of words”

**DOI:** 10.3389/fnins.2023.1080861

**Published:** 2023-02-09

**Authors:** Cedric Boeckx

**Affiliations:** ^1^Section of General Linguistics, Universitat de Barcelona, Barcelona, Spain; ^2^Institute of Complex Systems, Universitat de Barcelona, Barcelona, Spain; ^3^Catalan Institute for Research and Advanced Studies (ICREA), Barcelona, Spain

**Keywords:** *Homo sapiens*, deserts of introgression, symbol, innovation, imitation, self-domestication

## Abstract

This paper makes three interconnected claims: (i) the “human condition” cannot be captured by evolutionary narratives that reduce it to a recent ‘cognitive modernity', nor by narratives that eliminates all cognitive differences between us and out closest extinct relatives, (ii) signals from paleogenomics, especially coming from deserts of introgression but also from signatures of positive selection, point to the importance of mutations that impact neurodevelopment, plausibly leading to temperamental differences, which may impact cultural evolutionary trajectories in specific ways, and (iii) these trajectories are expected to affect the language phenotypes, modifying what is being learned and how it is put to use. In particular, I hypothesize that these different trajectories influence the development of symbolic systems, the flexible ways in which symbols combine, and the size and configurations of the communities in which these systems are put to use.

## 1. Framing: Beyond “modern” and “archaic/ancestral”

There is no shortage of books or papers addressing the question of “what makes us human?” This should not come as a surprise. As a species we have a remarkable ability to reflect on objects and events, and it is only natural to apply this ability to ourselves.

There is also a sizeable literature implicating “language” in the answer to the question of “what makes us human?” Again, this is not too surprising: language use lies at the center of much of what we do as a species. It is the most common currency for our daily social transactions. It makes sense to focus on it in trying to define who we are. Language looks to us pretty much like what the trunk is to the elephant, the neck to the giraffe, and echolocation to bats. Just like these other traits just mentioned, language may not be this entirely unique and antecedent-free capacity, as it is sometimes claimed to be (Anderson, 2004; Berwick and Chomsky, 2016), but it is certainly a very salient trait of our species.

Accordingly, I will be focusing on language in this Perspective, examining its nature with a view to shedding light on the “human condition.” I will do so from an evolutionary and interdisciplinary angle, inspired by the sort of question raised by Rutherford (2020) in another context (and with another focus). In an interesting thought experiment, Rutherford asks what would have happened to some arguments if, back in the eighteenth century we had known about the range of genetic facts now in our possession. It seems clear that many (bad) ideas would have been discarded from the get-go if such evidence had been available back when these ideas began to be formulated. We can ask the same question about the evolution of language and its contribution to the human condition: knowing what we know now about human evolution, and in particular having access to high-quality genomes of our closest extant and extinct relatives (Meyer et al., 2012; Prüfer et al., 2014, 2017; Mafessoni et al., 2020), are there positions along the spectrum of possible hypotheses regarding the evolution of language and cognition that we can now safely put aside as wrong or so implausible as not to be worthy of serious consideration?

I have argued elsewhere (Boeckx, 2017b, 2021; Martins and Boeckx, 2019; de Boer et al., 2020) that an entire class of evolutionary narratives exemplified by Berwick and Chomsky (2016), which posit one or a few key changes at the level of the genome and the brain that are claimed to have sparked a recent cognitive revolution in our lineage, have lost their initial conceptual appeal because the evolutionary trajectory of our lineage is clearly vastly much more complex than we used to think even just two decades ago. The twists and turns, booms and busts, carefully uncovered over the past decade in (geography/climate-aware) archaeology (Scerri et al., 2014, 2018, 2019; Groucutt et al., 2021; Kaboth-Bahr et al., 2021; Foerster et al., 2022; Gosling et al., 2022), alongside the numerous instances of gene flow across species that can be inferred from ancient genomes (Bergström et al., 2021), leave little room for doubt. There was no “great leap forward” (contra Diamond, 1991). Detailed comparative analyses of ancient genomes cataloging changes that set contemporary humans apart from neanderthals and denisovans (Pääbo, 2014; Kuhlwilm and Boeckx, 2019; McArthur et al., 2022), did not, and at this stage are highly unlikely to reveal “big bang” mutations of the sort Berwick and Chomsky (2016) appear to anticipate.

There is also another class of evolutionary narratives that one can set aside. This class of narratives is currently enjoying a fair amount of popularity in large part because of the demise of the scenarios discussed in the previous paragraph. Such narratives build on the evidence that questions recent “modern” origins for language and cognition only to conclude that such capacities were present in at least the last common ancestors we share with neanderthals and denisovans. Such approaches (Zilhão, 2011; Dediu and Levinson, 2013; Conde-Valverde et al., 2021) build on the renewed appreciation for the complexity of neanderthal behavior (Wragg Sykes, 2020), derived anatomy (D'Anastasio et al., 2013), as well as the presence of genetic fingerprints associated with features traditionally associated with “cognitive modernity” or enhanced cognition (Enard et al., 2002; Florio et al., 2018).

While such approaches undoubtedly capture an important fact—many “derived” aspects of our cognitive/behavior apparatus long preceded the emergence of our species—, I believe they suffer from some of the same limitations that afflict the implausible “recent great leap forward” scenarios discarded above. They fall into the same trap of simplistic scenarios: “if it's not very recent, it must be very old,” as it were. They also blindly adopt arguments used by “recent great leap forward” scenarios whose robustness can be said to be questionable. The “recent great leap forward” scenarios took virtually any discovery about cave painting, complex tool use, ornaments and the like to point to the recent emergence of cognitive modernity, because such discoveries were first made in a European context that fitted well with a simple, recent Out-of-Africa expansion. Crucially, though, the causal link between such discoveries and cognitive modernity was not made very explicit. They just looked like the sort of product of modern behavior, and since they appeared to be tied to “modern” human migration and settlement, it just made sense to treat them as reflexes of “cognitive modernity.” The evidence for this often rested on an argument from poverty of imagination, well illustrated in Berwick and Chomsky (2016): how could such behaviors be possible in the absence of (“modern”/complex) language (as we know it from contemporary use)? Now that at least some of these behavioral products have been attributed to neanderthals, the very same loose causal links are being used to ascribe cognitive modernity (and modern linguistic ability) to our closest extinct relatives.

Although proponents of this kind of “ancient cognitive modernity” scenarios often appeal to “the tyranny of the discontinuous mind” to argue against recent great leap forward scenarios (Zilhão, 2019), they in fact fall prey of the same tyranny of discontinuity (binarity): if it's not “recent *sapiens*,” it must be very ancient (“last common ancestor with closest extinct relatives”). In doing so, they ignore many possible nuances and qualifications that I think one must consider seriously in light of the fact that our lineage has a longer history than the “mere” 200,000 years that until recently was taken as the origin of us (Stringer, 2016). There is at least twice as much time, and therefore much room “in the middle,” so to speak, in order for evolution and natural selection to introduce and, more importantly, amplify differences.

Although it is fair to say that the past decade in archaeology has reduced the gap between closely related *Homo* species significantly, it has not completely eliminated differences. Indeed, it has produced more robust evidence for fine-grained differences. Some of these differences may be “neutral.” For instance, the morphology of ear ossicles in modern (contemporary) humans is different from that in neanderthals but this difference does not appear to have made a functional difference (Stoessel et al., 2016). Other differences seem to me to constitute sources of genuine (functional) differences. To list a few salient ones: facial differences associated with distinct behavioral affordances (Godinho et al., 2018; Zanella et al., 2019), braincase differences associated with differential expansion of specific brain regions (Gunz et al., 2010, 2019; Boeckx and Benítez-Burraco, 2014; Boeckx, 2017a; Kochiyama et al., 2018; Neubauer et al., 2018), differences in neural architectures and processes revealed by experimental manipulations (Stepanova et al., 2021; Trujillo et al., 2021; Mora-Bermúdez et al., 2022; Pinson et al., 2022), specific techno-complexes likely requiring specific cognitive skills such as bow-and-arrow technology (Lombard and Haidle, 2012; Lombard, 2019), earlier and more widespread use of ochre (Brooks et al., 2018; Sehasseh et al., 2021), therianthrop sculptures and paintings (Longa, 2013; Aubert et al., 2019), figurative art (as opposed to the more generic cave wall marks) (Hoffmann et al., 2018; Brumm et al., 2021), modified hand stencils suggestive of distinct communicative use (Etxepare and Irurtzun, 2021), and specific use of marks indicating a distinct numerical cognition beyond the widely shared number sense (d'Errico et al., 2018).

Some of the differences just enumerated may disappear in light of future discoveries, but I think it is unlikely that all of them will go away. Leaving them unexplained begs more questions regarding the cognitive structures they require. Note as well that unlike the “recent great leap forward” arguments of yore, there is no suggestion on my part that all these differences clustered in a short window of time, or that a single ability underlie them. In fact, concerning their timing of emergence, it appears that some differences emerged early in our lineage (associated with what Chris Stringer would call “basal” *Homo sapiens*), such as some facial features (Hublin et al., 2017), and others appearing later (associated with what Chris Stringer would call “derived” *Homo sapiens*) (Neubauer et al., 2018), consistent with the “mosaic”-like, piecemeal appearance of mutations that appear to be specific/derived in our lineage (Andirkó et al., 2022). As a matter of fact, this catalog of differences casts serious doubt on the once popular conundrum of “cognitive modernity,” with researchers seeking explanations for the gap between the emergence of anatomical modernity (back then dated at around 200ky) and behavioral modernity (around 50ky) (Sterelny, 2011). It is now clear that no such gap exists: both anatomical and cognitive “modernity” are the result of a temporally extended window of opportunities (as anticipated by McBrearty and Brooks, 2000). They are best thought of in terms of continuous dimensions instead of discrete states. Accordingly, “modernity” is best discarded as a concept.

It is also important to stress that highlighting differences between us and our closely related (extinct) relatives in no way implies any notion of superiority of our lineage. Extinct lineages no doubt exhibited derived traits of their own, and although they eventually got extinct, there is little evidence that this was caused by the direct effects of traits that may now characterize our lineage. Consider, for instance, the fact that between 150,000 and 350,000 years ago, the Y chromosome of the Neanderthals was totally replaced by that of a *sapiens* population (Petr et al., 2020), following introgression (Kuhlwilm et al., 2016). This likely impacted the evolutionary trajectory of the neanderthal lineage, considering that neanderthals were already experiencing a reduction in population size (Mafessoni and Prüfer, 2017; Skov et al., 2022). Crucially, though, this replacement-post-introgression event (or events) implicated (at least) one *sapiens* population that itself got extinct (as did others, which did not displaying the full suite of anatomical “modernity” Harvati et al., 2019; Prüfer et al., 2021). So, even if one population impacted another, both got extinct. The extinction of *Homo* lineages is likely the outcome of a complex mixture of environmental factors, with the influence of “modern” lineages probably more indirect than previously thought. It is quite likely that different causes were responsible for the extinction of distinct *Homo* populations, which were more heterogeneous than cover terms such as species names tend to suggest.

Demography is in fact frequently mentioned as the most likely cause of extinction of *Homo* lineages (Wragg Sykes, 2020; Vaesen et al., 2021). This offers me another opportunity to question currently popular scenarios about the evolution of cognition and language. Recall that these (rightfully) abandon recent-great-leap-forward scenarios to jump to the conclusion that “recognizably modern language is likely an ancient feature of our genus pre-dating at least the common ancestor of modern humans and Neandertals about half a million years ago” (Dediu and Levinson, 2013). Curiously, this argument makes more sense in the context of views about the nature of the language faculty that are at the heart of recent-great-leap-forward scenarios, such as Chomsky's, which assumes a strong, domain-specific genetic component structuring our language capacity. Other perspectives on language favor a view where weak (generic) cognitive biases (rooted, eventually, in our genomes) have significant, detectable effects on behavior only in the context of social/demographic differences (Thompson et al., 2016; Raviv, 2020). These views predict important differences in language development and behavior among *Homo* lineages (currently not the consensus view) if demographic/social differences exist between these (which is the consensus view), because (to put it in terms of Hurford, 1990) the “arena of use” shapes the “language acquisition device.”

My own position is indeed that precisely because such social differences existed, important cognitive and behavioral differences must have existed, and we can understand the nature of these better by tracing down the biological roots of social differences, examining ancient genomes and asking about the neural consequences of a wide range of mutations. True, doing this requires abandoning a traditional dichotomy between “nature” and “nurture”/“culture” or between “social”/“cultural” and “cognitive,” but there is a lot of evidence that such dichotomies are spurious anyway.

As eloquently discussed by Thomas (2014) and Thomas and Kirby (2018), cultural accounts, whose plausibility is on the rise, cannot, on their own, be the whole story. For them to come into effect, they require preconditions that depend at least in part of the biology of users (pretty much like a niche that requires construction by its users). As Thomas (2014) puts it, “cultural accounts cannot constitute a full alternative to biological accounts until they are paired with an explanation of how such preconditions themselves are possible.” In the context of language, as Thomas and Kirby (2018) remark, “if cultural evolution can account for language structure, …we face the task of accounting for the origin of the traits that enabled that process of structure-creating cultural evolution to get started in the first place.” This is, as they note, radically different from thinking of language structure “as reflecting an accumulated set of changes in our genome,” where the question is “What are the genetic bases of language structure and why were they selected?” (cf. Pinker and Bloom, 1990). Instead, under cultural accounts, the focus is put on the biological underpinnings of the cultural process. To put it in terms of Heyes (2018)'s hypothesis of domain-specific cognitive abilities being in fact “cognitive gadgets” made possible by culture, a central question becomes “What are the biological underpinning of the factory eventually responsible for the manufacturing of cognitive gadgets?”

As anticipated by Hurford (1990) over three decades ago, the “arena of use” acquires special significance in this context, and it is the process of construction of distinct ecologies that I focus on in the rest of this paper in an attempt to characterize what made us “hunter-gatherers of words,” that is, distinct language users (individuals that acquire/hunt words and use/gather them flexibly). Accordingly, my hypothesis falls under the rubric of usage-based accounts.

## 2. Signals from comparative (paleo)genomics

My starting point to gain insight into the underpinnings of socio-demographic differences across closely related species comes from evidence in favor of a hypothesis that has gained traction in recent years, the “self-domestication” hypothesis (Hare, 2017; Theofanopoulou et al., 2017; Wrangham, 2019; Zanella et al., 2019; Boeckx et al., 2022; Spikins, 2022). Part of the reason this hypothesis is currently popular stems from the possibility of identifying deep homology and convergence of molecular mechanisms between canonical domesticated species like dogs and species claimed to have undergone a similar process of reduction in reactive aggression (possibly coupled with, or followed by a neurocristopathic process). Such a possibility to test a hypothesis arises in the context of analytic options afforded by the availability of ancient genomes, which has not only revolutionized our understanding of our lineage's deep past, but also that of domesticated species (Frantz et al., 2020).

Initial work along these lines (Theofanopoulou et al., 2017) highlighted the role of neurotransmitters. This received additional support from a systematic examination of more species and a more detailed characterization of the nature of neurotransmitters associated with signals of positive selection (O'Rourke and Boeckx, 2020). This study looked at genomic changes and gene expression differences among 488 neurotransmitter receptor genes across 14 domesticated species and found that the most convergent signal of selection across species exhibiting reduced reactive aggression is associated with genes implicated in glutamate signaling. Specifically, kainate and two groups (II and III) metabotropic glutamate receptor genes showed disproportionately high rates of changes. Importantly, as emphasized in O'Rourke et al. (2021), these receptors are distinct from other glutamate receptors in that their actions tend to downregulate glutamate release, affecting stress circuits in specific ways. By hypothesis, changes associated with these genes underlie the dampening of excitation in the relevant neural circuits controlling reactive aggression (O'Rourke and Boeckx, 2020).

Here I will take these results to be essentially valid, and explore a question that I begin to address with colleagues in O'Rourke et al. (2021), where we argued that the changes to genes involved in regulating glutamatergic signaling can not only account for the core reduction in reactive aggression underlying domestication, they have additional effects, such as modifications of how motor output for both reactive aggression and learned vocalization interact. If correctly characterized, such effects could account for vocal production differences between closely related vocal learning species like the (domesticated) Bengalese finch and the (wild) white-rumped munia.

A reduction in levels of reduced aggression is in fact known to be accompanied by other traits, for instance reduced neophobia, or indeed neophilia (Suzuki et al., 2021). Given the significant differences in gene expression in brain regions involved in learning, memory, and executive function between neophobic and non-neophobic populations (of, e.g., house sparrows, Lattin et al., 2022), especially in glutamate-signaling genes already highlighted in O'Rourke and Boeckx (2020), it is reasonable to expect “major differences in neural function …that could affect a wide variety of behavioral traits beyond neophobia” (Lattin et al., 2022).

In fact, a close comparison (Audet et al., 2018) of wild-caught individuals from two species that are close relatives of Darwin's finches and that differ in problem-solving skills, found robust differences in glutamate receptor expression reminiscent of differences between domesticates and their wild counterparts (Wang et al., 2018; O'Rourke and Boeckx, 2020). In particular, the GRIN2B/GRIN2A ratio, known to correlate with synaptic plasticity, was higher in the more innovative finch species. Interestingly, the GRIN2B/GRIN2A ratio is know to change over the course of development (Pegasiou et al., 2020). We reported a similar age-dependent effect is observed in the context of domestication (Eusebi et al., 2022). This effect may be related to exploration and exploitation shifts over development (Gopnik, 2020; Liquin and Gopnik, 2022), and may bear on the general rubric of neotenic traits (protracted childhood) long associated with self-domestication (Wrangham, 2019), although the concept of neoteny is likely to be too broad and general to be useful in capturing the patterns under discussion.

Intriguingly, in the context of this discussion, genes found in the most restrictively defined lists of deserts of introgression in modern human genomes (regions of the genome depleted of introgressed alleles from neanderthals/denisovans) (Reher, 2021), such as *CADM2*, have been linked to personality traits like risk-taking behavior, impulsivity, addiction (Pasman et al., 2022; Sanchez-Roige et al., 2022). (Similar signals have been associated with other genes in introgression deserts such as *CADPS2* or *FOXP2* Sanchez-Roige et al., 2022). Of note, the gene (*CADM2*) is listed alongside glutamate receptor *GRIK3*, among the top candidate genes with potential connections to behavior in domestication studies (Freedman et al., 2016). *KCND2* is one of a handful of genes found in introgression deserts that are associated with signals of positive selection (Sankararaman et al., 2016; Vernot et al., 2016; Chen et al., 2020; Reher, 2021; Buisan et al., 2022), and also exhibits strong association with cognitive flexibility (Hu et al., 2020).

The presence of these genes in introgression deserts is of great interest, as these regions are relatively few, and, in part because of this, are often mentioned as plausible entry points into some of the most distinctive aspects of the human (“*sapiens*”) condition. Mutations affecting the genes in these regions may underlie behavioral/personality-type/temperament differences (in the sense of Réale et al., 2007) between closely related species, the more so because some of these deserts only purged introgression in a unidirectional manner among hominins (Kuhlwilm, 2018). Such elimination of introgressed alleles in specific regions of the autosomes may be linked to evidence of increased childlessness being mediated by genetically associated cognitive and behavioral traits (Gardner et al., 2022).

It is also interesting to note that the expression of these genes in the brain is associated with significantly different developmental trajectories in only a few regions, most saliently the cerebellum (Buisan et al., 2022) (see also Andirkó and Boeckx, 2022; Andirkó et al., 2022), and for that region, with evidence of heritability depletion measures in just one lobule (Crus II) (Carrion-Castillo and Boeckx, 2022), most frequently implicated in complex socio-communicative functions (Van Overwalle et al., 2020; Nakatani et al., 2022). This may ultimately related to claims of enhanced turn-taking in our species (Kendrick et al., 2020; Levinson, 2022), with effects on language use.

Behavioral phenotypes similar to those associated with *CADM2*-variation (impulsivity, risk-taking behavior) mentioned above are also reported with variation linked to serotonin, another neurotransmitter frequently brought up in domestication studies (Wang et al., 2018). Specifically, Kameneva et al. (2022) posit that chromaffin cell number (and adrenal gland size) control via serotonin-sensitive precursor (co-called “bridge”) cells, which also express *GRIK3* and *CADPS2* already mentioned above, “may provide a regulatory serotonin-mediated pathway of prenatal programming for long-lasting changes in progeny underlying the behavior of domesticated species as well as wild animals with active and reactive types of coping strategy.” As Kameneva et al. (2022) note, “[a]ggressive males typically express a more proactive type of behavioral response demonstrating rigid, cue-independent, and impulsive reactions and a tendency to defend their home territory. …Non-aggressive reactive males are rather flexible, cautious, and open to the external cues, which can assist in variable or unpredictable environments.”

Incidentally, many of the genes highlighted in this section are among genes under positive selection in a comprehensive study of the genetics underlying domestication in laboratory (classical inbred) mice (Liu et al., 2022), indicating again the likely behavioral consequences associated with these gene variants.

The presence of *FOXP2* in the large introgression desert on chromosome 7 may have less to do with its well-known role in sensori-motor learning, and more to do with its associations with behavioral traits pertaining to sociability and externalizing behavior (Tielbeek et al., 2022; Verweij et al., 2022).

Coping with variable or unpredictable environments has long been seen as a defining feature of our lineage. It is the basis of the “generalist specialist niche” hypothesis (Roberts and Stewart, 2018), put forward to account for our species' unique ecological plasticity. This ability is likely to have developed thanks to a social characteristic that is an explanatory target of self-domestication-based accounts (Hare, 2017; Hare and Woods, 2021): our ability to cooperate with other *Homo sapiens* to whom we weren't related. Proponents of such accounts repeatedly invoke oxytocin as central to this process. It is interesting in this context that in an attempt to define its role beyond social dimensions, Quintana and Guastella (2020) define oxytocin as an allostatic hormone that modulates both social and non-social behavior by maintaining stability through changing environments, which is a property one expects from generalist specialists. Although oxytocin did not figure prominently in our initial attempt to identify convergence across domesticates (Theofanopoulou et al., 2017), our subsequent comprehensive examination of oxytocin/vasotocin receptors in the evolution of our lineage identified a unique pattern of convergent evolution in modern humans and bonobos (Theofanopoulou et al., 2022). It is also worth pointing out that several genes associated with signals of positive selection and found in deserts of introgression, such as *ROBO2* and *CADPS2*, have been associated with the release of oxytocin (Anbalagan et al., 2019; Fujima et al., 2021).

All in all, it seems to me that signals from regions of the genome under positive selection and/or depleted of introgressed alleles from our closest extinct relatives point toward derived variants in our lineage having had significant social effects accompanied by secondary behavioral traits that collectively point to differences between species (though of course this remains to be tested experimentally). Thinking along the lines of Legare and Nielsen (2015), who view imitation and innovation as the dual engines of cultural learning (see also Jagiello et al., 2022; Whiten, 2022), I'd like to put forward the hypothesis that the variation in our genome briefly reviewed in this section contributed to shifting the balance between these two engines and promoted a “personality” type favoring innovation/exploration over imitation/exploitation. These are of course continuous dimensions, and we should not expect binary types here. Instead, we can think of them as two factors constituting the two principal components of a complex, multi-dimensional space.

My suggestion is very much in line with proponents of the “Cultural Intelligence” hypothesis, which claims that more frequent opportunities for social learning should boost an individual's repertoire of learned skills (Van Schaik and Burkart, 2011; Forss et al., 2016; Schuppli et al., 2017; Forss and Willems, 2022). Put differently, improved social learning should boost asocial learning (and in general, a reciprocal causality pattern between biology and culture, with each imposing selective forces on the other, Whitehead et al., 2019). Evidence that early sociability fosters later exploratory tendency (“curiosity”) has come from closely related primate species such as *Pongo abelii* and *Pongo pygmaeus* (Schuppli et al., 2020). Interestingly, genomes of these species reveal marked differences in signals of adaptive evolution (Mattle-Greminger et al., 2018) implicate some of the very same regions that are under positive selection and/or depleted of introgressed alleles from other hominins. In particular, the genomes of more prosocial and innovative Sumatran orangutan (*Pongo abelii*) show multiple signs of convergence for positive selection with our lineage and some domesticated species (original observations I owe to unpublished work by Sara Silvente i Font).

My suggestion is also in line with the claim that social differences may be enough to capture cultural differences, without specific biological adaptations for cultural transmission (Scott-Phillips, 2022).

## 3. Linguistic/cognitive phenotypes

One of the virtues of the cultural intelligence hypothesis is that, just as Thomas and Kirby (2018) encouraged researchers to do in the context of language evolution, it specifies the conditions in which benefits of a boost in intelligence can be reaped. In the present context, these conditions include a shift toward great exploration, a consequence of reduced reactive aggression (“self-domestication”). As such, my hypothesis departs from the role ascribed to self-domestication in Thomas (2014); Thomas and Kirby (2018), where self-domestication is said to set the stage for the cultural transmission process responsible for language evolution. Building on previous work (e.g., Kirby et al., 2015), Thomas and Kirby (2018) view the process of structure-creating cultural evolution as requiring two key precursor traits at the heart of which they place self-domestication: (i) the transmission of the communication system through learning; and (ii) the ability to infer the communicative intent associated with a signal or action.

While I agree with them that these are essential ingredients of the cultural process, I do not think self-domestication was critical in their emergence, for they strike me as too generic and widespread in the animal kingdom. It is true that their effects have been studied with animals in (“artificial”) experimental settings that may make one think of human influence and domestication (Fehér et al., 2009; Claidiere et al., 2014), but it is also possible to find them in the wild (Whiten, 2019, 2021; Williams and Lachlan, 2022; Williams et al., 2022). Thomas and Kirby (2018)'s intuition that self-domestication affected the cultural process strikes me as being on the right track, but the effect we should be looking for must be more specific than they claim. For me, it is not the emergence of the two principal components of the cultural transmission process that is the answer. I think that, like many other components of human language and cognition, these were in place well before the origin of our lineage, as the refinement of stone tools suggest (Stout and Hecht, 2017; Shipton, 2019), which certainly required sustained knowledge transmission over generations, and changes in sociability, with particular importance given to conformity (Raghanti et al., 2018). Instead, it was a shift in the way in which learning and communicative intent interact, with communicative intent gaining the upper-hand, as it were. In particular, what expanded, in my view, is the tendency to expand and explore what Raviv et al. (2019) refer to as the “meaning space” (novel meanings).

This hypothesized shift seems to me to be sufficient to capture the intuition underlying Derex (2022)'s distinction between Type I and Type II cumulative culture. Derex defines Type I as a process optimizing cultural traits that exploit a given set of natural phenomena (which he takes to be potentially widespread in nature), and the much more specific (possibly, human-specific) Type II, which is the process expanding the set of natural phenomena we exploit. Type II pretty much corresponds to the effect I attribute here to the self-domestication in our lineage. If I am right, we do not need to posit two distinct types of cumulative cultural evolution. The difference between Derex's Type I and Type II reduces to distinct values along the principal components that account for cultural transmission.

The role I ascribe to self-domestication is quite possibly what van Schaik et al. (2019) call “true” cumulative culture, and in particular the “steep increase in cumulative culture …reflect[ing] the rise of active novelty seeking (curiosity), which led to a dramatic range expansion and steep increase in the diversity and complexity of material culture.” van Schaik et al. (2019) take the emergence of teaching (akin to natural pedagogy, Csibra and Gergely, 2009) to have been critical to this process (see also Rutherford, 2018). I am less convinced by this, because what I think mattered more is the sort of deferral seen in learning, rather than the dominant teaching relation. Work on domesticates (Range et al., 2019) has shown that domesticates cooperate in a way distinct from their wild relatives, with the latter adopting a more asymmetric, dominant relation (reminiscent of “teaching”), and the former displaying a more submissive/tolerant relation (akin to “learning”). Interestingly, as Kirby and Tamariz (2022) have shown, learning from learners impacts cultural transmission, speeding up the process considerably. In related work, Raviv et al. (2019) show that increasing the number of interaction partners can replace the need for multiple generations of learnings and shape the formation of linguistic structure. This has the advantage of making the process of cumulative cultural transmission more robust, as it makes it less dependent on the maintenance of skills over generations that rely specifically on a few individuals that are experts-teachers (Thompson et al., 2022). The latter setting is much more fragile, and subject to environmental conditions, endangering the long-term maintenance and benefits of the “ratchet effect” of cumulative culture (a situation that may have characterized the neanderthal condition and evolutionary trajectory, Power et al., 2013). (For relevant discussion, see also Cantor et al., 2021).

At this point I'd like to examine more closely the role self-domestication may have played in shaping our communicative system by shifting the relation between exploration and exploitation toward the former. I think the reason our communicative system gained in complexity (in the sense of Chomsky, 1956) rests on an important innovation that set the stage for others: words, i.e., symbolic units that not only have reference (like icons and indices have), but also, and crucially, sense. The “special” nature of human words has been well captured by Chris Knight in a series of works (Knight et al., 1995; Knight, 2008, 2010). By being truly symbolic, words are “patently false signals.” They are not mere fictions (but like fiction, they require a special, safe ecology to prosper, Dubourg and Baumard, 2022). They are facts, but “facts whose existence depends entirely on subjective belief” (Knight, 2010). In Searlian terms (Searle, 2010), they are “institutional facts”: fictions that are granted factual status within human social institutions. As Knight observes, “linguistic utterances are symbolic to the extent that they are patent falsehoods serving as guides to communicative intentions” (Knight, 2010). They are communicatively useful untruths, as it were.

The reason words could be that way is, as Knight (2010) insightfully remarks, largely down to a matter of trust. In his words, “language began to evolve when humans started reciprocally faking in communicatively helpful ways,” i.e., whey they became “capable of upholding the levels of trust necessary for linguistic communication to work.” Knight (2010) is correct that animal must always carry at least some of the burden of generating the trust necessary for communication to work. That is, the signal must be connected to tangible “truths of the matter” to be trusted, which I understand to mean it must be either iconic or indexical (the two types of signs associated with their referents in C.S. Peirce's well known typology). As Knight (2010) points out, if trust can be taken for granted, then it leaves the signaller free to concentrate only on perceptual discriminability. “Carried to its conclusion, this should permit digital signaling—the cheapest and most efficient kind of communication.” For Knight, animal signaling cannot be digital because it doesn't have the luxury of being patently false or fictional. “Costly signals of any kind can only be evaluated on an analog scale.” Put differently, truly symbolic, digital signals “are acceptable only under highly unusual conditions—such as those internal to a ritually bonded community whose members are not tempted to lie” (Knight, 2010).

For me, self-domestication, by reducing levels of reactive aggression, that is, lowering levels of fear, and boosting levels of trust, created just the right context for symbolic communication to be stable (in an ever-expanding community, which is another consequence of trust). It made it possible for words to be detached from the bounds of reality (truth, reference), thereby allowing for human language to achieve a level of flexibility and creativity (world-making) that makes our system of communication and thought quite special. In other words, self-domestication created an ecology that made it possible for users (learners, communicators) to abandon the need to rely on “credible signaling” (an important function of past communicative systems, Mehr et al., 2021), in effect allowing them to suspend their disbelief in the face of “honest fakes,” and setting the stage for the strongest meaning of “arbitrary” signals in the sense of Planer and Kalkman (2020) (see also Gasparri et al., 2022; Watson et al., 2022). I thus concur with Rossano (2010) that social factors were critical in the evolutionary emergence of symbolism, but disagree with him that symbols impose special cognitive demands. Rather, they demand a special ecology, not available to our closest extinct relatives, which fits with claims that rituals meant something different for them (Nielsen et al., 2020), and that ornaments were indexical in the Peircean sense (Rossano, 2010). (For relevant discussion, see also Tomlinson (2018), where our closest extinct relatives are associated with stages of (hyper)indexicality, but not symbolism.)

Of course, the adoption of truly symbolic tokens of communication does not imply that iconic and indexical traits were banished from language. Such properties are extremely useful as trust is being built in the course of language acquisition (Imai and Kita, 2014), and their presence in modern languages indicate that they continue to fulfill an important supportive role in communication (Perniss et al., 2010; Cuskley, 2013; Perlman and Cain, 2014; Perlman et al., 2015; Filippi, 2016; Perlman, 2017; Motamedi et al., 2022). But still, words *qua* symbols may have shifted the balance between different modalities at work in language, giving more prominence perhaps to the vocal modality, which is particularly amenable to an organization captured by the concept of “duality of patterning” (De Boer et al., 2012), compared to other modalities that have stronger iconic connections. Duality of patterning certainly contributed to the explosion of vocabulary, and set the stage for subsequent syntactic exploration, relying on endogenous attention (Irurtzun, 2015; Martinez-Alvarez et al., 2017; Orpella et al., 2020). Such exploration may well have taken the form of reliance on variable binding (what some syntacticians would refer to as “internal (use of) merge,” or “abstraction,” Pietroski, 2018 and Icard and Moss, 2022), allowing for representation whose format suits what Bolender (2007) calls “cognition by description,” which marked a further departure from the limitations imposed by knowledge (cognition) “by acquaintance.” In this sense, symbols were the true springboard for our algebraic minds (Marcus, 2003). In this sense, words and syntax are linked, and the crucial relation is semantic use (pragmatics in a broad sense).

There is a parallel to be made at this point between the evolutionary scenario advocated here and the evolution of song complexity (variability) in the domesticated Bengalese finch studied by Kazuo Okanoya over the years (Okanoya, 2004, 2017). Okanoya posits multiple stages and selective pressues leading to the range of songs characteristic of Bengalese finches (compared to those of wild white-rumped munias). Specifically, he posits a first stage of domestication, relaxing constraints, which set the stage for a subsequent stage of (sexual) selection promoting song complexity within the Bengalese finch lineage. Such multi-step scenarios are also considered in the history of other domesticates (Boeckx et al., 2022; Herbeck et al., 2022). In the case of language evolution and change, they may correspond to the establishment and maintenance of norms or conventions (Hawkins et al., 2022), distinguishing communities of language users (Iacozza et al., 2019), in which, in Goldberg (2019)'s terms, speakers balance between Expressiveness (communication) and Efficiency (compression) while conforming to conventions, resulting in the pervasive phenomenon of “partial productivity.”

In closing this section, I'd like to point out that if the argument put forward here is on the right track, it suggests that Wrangham (2018, 2021)'s conjecture that language was critical for the emergence of self-domestication in our lineage cannot be right. It is the reverse, in fact (in line with Thomas and Kirby, 2018's intuition): self-domestication was crucial to the development of central traits of our communicative system. Of course, cooperation and communication are linked, but there is evidence that the coevolutionary relationship between vocal communication and group-level cooperation is not unique to humans (Mine et al., 2022). Instead, the factors that facilitated self-domestication in our lineage must be looked for elsewhere. For instance, the founder sociality hypothesis (Brooks and Yamamoto, 2021) proposes that the social dynamics of founder populations in novel and newly available environments can have critical effects in shaping species' sociality and can produce long-lasting changes in social structure and behavior. As Brooks and Yamamoto (2021) write, “[f]or founder populations which expand into an underexploited niche separated from the parent population, the necessity of bond formation with strangers, lack of clear territories, and initial abundance of resources can lead to altered initial social dynamics” impacting subsequent evolutionary trajectories. Prevalence of specific dominance patterns in the dispersing group may also have played a role. There is evidence for fewer aggressive acts being used in societies where female dominance prevails (Davidian et al., 2022; Kappeler et al., 2022), as a result of sex differences in reactive aggression (Aubry et al., 2022). This line of thought would fit well with reverse dominance patterns hypothesized by Knight et al. (1995); Knight (2010), building on Boesch (2009), to account for the earliest functions of ochre-based symbols in our lineage (Potts et al., 2018, 2020).

## 4. Conclusion

Competing narratives about language evolution continue to be framed in binarity terms (Hauser et al., 2002), looking for neural underpinnings that gave us an edge (Berwick and Chomsky, 2016), or failing that, concluding that our “modern” language faculty may be an ancestral trait (Dediu and Levinson, 2013). Such stances illustrate one of the most pervasive fallacies in evolutionary thinking about language and cognition (Fujita, 2016).

Here I have argued that there is a lot of evidence to suggest that a substantial fraction of the neurological and anatomical underpinnings of linguistic cognition were in place well before the emergence of the *sapiens* lineage. In this set I include important traits like joint intentionality (Tomasello, 2019), the neurobiological basis of “dendrophilia” (Fitch, 2018), and loss of complexity in vocal anatomy (Nishimura et al., 2022). But such foundations were subsequently modified in the course of our lineage's (extended) evolution (Stringer, 2016; Scerri et al., 2018), during which cognitive biases already in place were differentially mobilized, giving rise to distinct ecologies, with different cognitive biases being recruited and forming clusters. In particular, on the basis of results from comparative analysis of (ancient) genomes, I have hypothesized that a process of self-domestication (reduction in reactive aggression) had the net effect of tilting the balance between exploratory vs. exploitative personality types in favor of the former, relaxing constraints that led to significant innovations in the architecture of the language faculty and cognition, including truly collective intentionality (Tomasello, 2019), symbolic culture (Knight, 2010), and mental representations supporting cognition by description (Bolender, 2007).

The net effect of these modifications is an increased reliance on culture. We are a truly enculturated species, and our language (both in shape and use) is perhaps the best reflection of the scale of cumulative cultural evolution in our lineage. But contrary to the consensus view, well articulated in Laland and Seed (2021), symbolic language is not this extra component that “underpin humanity's uniquely potent capacity for cultural learning” alongside “an unusually accurate, and intention-oriented, capacity for imitation” and “a generalized capacity for teaching and other subtle forms of information donation.” Symbolic language is in fact the result of particular values (parameters) along the two orthogonal dimensions of learning (compression) and information sharing (communication). These values end up giving rise to distinct ecotypes among closely related species, such as us and the neanderthals, but by no means limited to the *Homo* lineage (Hager et al., 2022; Vilgalys et al., 2022). Such ecotypes are associated with distinct fitness benefits despite high levels of gene flow.

If I am right, the history of the “modern” language faculty and the overall shape of our cognitive phylogeny is as complex and reticulate as what we now know about “modern humans” (*Homo sapiens*). The equation “sapiens” ≈ “loquens” is preserved but not in the way that it is traditionally articulated (Chomsky, 2015): the special character of our language system has less to do with the presence of a unique mental organ or neural circuit and more to do with the way it is put to use. It has less to do with learning to speak (developing a mental module), and more to do with speaking to learn (putting our system of communication to special use). If true, this means that distinctions like “modern language faculty” vs. “protolanguage” may not be so useful after all, and that gaps between “anatomically modern” and “cognitive modern” may be illusions. If the key to our success is characterized as flexible cooperation among large numbers of (unrelated) individuals (Harari, 2014), it is rooted in our reduced reactive aggression and the trust it generated, which enabled us to suspend our disbeliefs and elaborate symbolic systems. Words are indeed central.

## Data availability statement

The original contributions presented in the study are included in the article/supplementary material, further inquiries can be directed to the corresponding author.

## Author contributions

CB: conceptualization, formal analysis, writing, and funding acquisition.
